# The Apotheosis of Kipps

**Published:** 1919-04-05

**Authors:** 


### THE APOTHEOSIS OF KIPPS.

To be an inmate of an Air Force hospital in these closing months of the war is to be a fragment of wreckage thrown up on a beach after a tempest. Around are other fragments, some lightly scarred, some battered almost out of recognition. And of those craft which are still seaworthy and doing good service, scarce one but shows the traces of heavy weather. Matron has a Mons ribbon on her breast, Sister the Royal Red Cross, and even the junior V.A.D.s are saucy in Maltese and Imbros Greek.

As for us broken fragments, lying in the little beds, we are from the ends of all the earth. For when the Air Force started its huge expansion after the first three years of war it swept all manner of queer fish into its net. Prudent and reckless, sound and maimed, airmen and architects, baronets and butchers, chemists and clockmakers, dukes and drapers. . . . And so Kipps got his chance.

One has been a good deal troubled in mind about Artie Kipps during this war. One could rely on the little chap's patriotism, but somehow I couldn't see him happy as a soldier. He seemed too wedded to this established muddle we call civilisation to feel happy under a rough-tongued instructor in the arts of murder. I met him a few times in the ranks at the beginning, and was glad to see that the daily pound of beef which was then the Army ration was having a tonic effect. He filled out daily, became fat and cheery, developed impudence even. His new-found muscles let him play with his rifle as once he had trifled with his yard stick, and Army discipline was child's play after his experience of the rules of a drapery establishment. His wits were always alert, the work appealed to him, and he felt himself a man, which was entirely good for him, because he was too well trained ever to forget other people's feelings in his own growing egoism. He was always a little gentleman, was Artie Kipps, and being cheerful and obedient he became a very fine little soldier, and fought (albeit with a sick horror of the beastly details of bloodshed) and died a very gallant little man. So that I feel confident that, though dead, he died with the Right Vision before him.

Alive, I wasn't so sure. It's been a long war, and peace a weary, weary time a-coming, and stouter hearts than Kipps' have faltered by the way. But now I have met him again here, and am rejoiced to be able to report that all is well with the lad. He has the next bed to mine, and the coat he pulls over his shoulders when he limps down the long ward has the crowned albatross and ring of a second lieutenant on its sleeve. He won't fly again. One of his legs is five inches shorter than the other, and he will never bend his right elbow again, so that he can no longer reach the rudder-bar or manage the controls. But what do these trifles matter? He is a knight, proven and tried. The dreams he had in the dormitory at school have come true. Do you think future unemployment can bother an officer who has had an aeroplane and air mechanics of his own? a man who has met the enemies of his country ("dirty 'Uns") in the clouds, and fought with them and killed them? a man who has fallen six thousand feet with four wounds, and brought his bullet-riddled machine to earth with never a strained stay? who could give coherent directions to his mechanics before fainting on the way to hospital?

Once Artie Kipps feared troublesome customers, feared whilst envying his shop-walker, feared above all the dreaded "sack." Now he fears nothing on earth. He has outlived all that fear can do to him. Life henceforth is a great jest. From the moment he wakes in the morning till the Night Sister tells him to "Be quiet, Mr. Kipps," he is the life and soul of the ward. He confesses he is "full o' beans to-day, ol' bean." He has quit himself like a man—like a superman. So what can the future hold to make him fear?

